# Real-World Diagnostic Yield and Longitudinal Dynamics of Fluorescence In Situ Hybridization in Hematologic Malignancies: A Five-Year Retrospective Analysis of 1357 Patient Samples

**DOI:** 10.3390/genes17080955

**Published:** 2026-08-14

**Authors:** Rosalba Fumo, Katia Scala, Sara Gaeta, Angela D’Ardia, Teresa Infante, Giuseppe Ciancia, Alessandro Caputo, Alessandra Rosati, Bianca Serio, Antonio D’Antonio, Pio Zeppa, Massimiliano Chetta

**Affiliations:** 1 Complex Operative Unit of Pathological Anatomy and Molecular Diagnostics, University Hospital “San Giovanni di Dio e Ruggi d’Aragona”, University of Salerno, Largo Città di Ippocrate, 84131 Salerno, Italy; rfumo@libero.it (R.F.); katia.96.ks@gmail.com (K.S.); sara.gaeta@sangiovannieruggi.it (S.G.); adardia@unisa.it (A.D.); teresainfante84@gmail.com (T.I.); giuseppe.ciancia@sangiovannieruggi.it (G.C.); alessandro.caputo94@gmail.com (A.C.); pzeppa@unisa.it (P.Z.); 2 Specialization School in Clinical Pathology and Clinical Biochemistry, Department of Medicine, Surgery, and Dentistry “Schola Medica Salernitana”, University of Salerno, Via Salvador Allende, 84081 Baronissi, Italy; 3 Department of Medicine, Surgery, and Dentistry “Schola Medica Salernitana”, University of Salerno, Via Salvador Allende, 84081 Baronissi, Italy; arosati@unisa.it; 4 Hematology and Transplant Center, University Hospital “San Giovanni di Dio e Ruggi d’Aragona”, University of Salerno, 84131 Salerno, Italy; bianca.serio@sangiovannieruggi.it; 5 Department of Pathology, PO del Mare, Via Enrico Russo, 80147 Napoli, Italy; ada66@inwind.it

**Keywords:** fluorescence in situ hybridization (FISH), hematologic malignancies, cytogenetic abnormalities, clonal evolution, nomogram, real-world evidence

## Abstract

Background/Objectives: Fluorescence in situ hybridization (FISH) is an established component of the diagnostic and prognostic workup of hematologic malignancies. However, most evidence supporting FISH interpretation originates from treatment-naïve diagnostic cohorts, providing limited insight into how cytogenetic findings evolve during longitudinal disease monitoring and therapeutic exposure. We retrospectively analyzed 1357 consecutive FISH assays performed in 1092 patients with hematologic malignancies between 2019 and 2023. Methods: Multivariable logistic regression identified predictors of aberration detection, while longitudinal analyses explored temporal changes in cytogenetic profiles among serially monitored patients. Observed abnormality rates were compared with published diagnostic benchmarks. Results: The cohort had a median age of 68 years and was predominantly composed of patients with myelodysplastic/myeloproliferative neoplasms (27.2%), multiple myeloma (26.9%), and chronic lymphocytic leukemia (21.6%). Overall, 29.2% of informative assays revealed cytogenetic abnormalities. Clinical indication was the strongest predictor of aberration detection. Compared with diagnostic samples, follow-up specimens showed significantly lower odds of abnormal findings (adjusted OR 0.48, 95% CI 0.36–0.64; *p* < 0.001), whereas relapse samples demonstrated higher odds (adjusted OR 1.82, 95% CI 1.28–2.59; *p* = 0.001). Increasing age was independently associated with abnormal FISH results (adjusted OR per decade 1.24, 95% CI 1.12–1.38; *p* < 0.001). Several canonical abnormalities occurred less frequently than expected from historical diagnostic series, including BCR::ABL1 within the chronic myeloid leukemia testing pathway and del(13q14) in both chronic lymphocytic leukemia and multiple myeloma. In contrast, del(17p), involving the *TP53* locus, remained consistently represented across disease categories and was enriched in chronic lymphocytic leukemia relative to published diagnostic cohorts. Conclusions: Longitudinal assessment revealed increasing cytogenetic heterogeneity over time, with common lesions progressively declining and rare or complex abnormalities expanding from 16.3% to 36.7% of the observed cytogenetic landscape. The cytogenetic patterns detected by FISH in contemporary hematology practice are strongly associated with clinical context, disease stage, and treatment history; however, the cross-sectional and retrospective design precludes inference of causal influence. The divergence between real-world positivity rates and historical diagnostic benchmarks highlights the need for context-specific interpretation of cytogenetic findings. The persistence of TP53 loss across disease boundaries and the progressive diversification of aberrations over time underscore the dynamic nature of clonal evolution in hematologic malignancies.

## 1. Introduction

The introduction of fluorescence in situ hybridization (FISH) into hematopathology practice represented a fundamental conceptual change, redirecting diagnostic attention from metaphase chromosomes to interphase nuclei and enabling the detection of cryptic translocations, submicroscopic deletions, and copy-number alterations that constitute integral components of the World Health Organization classification of hematologic neoplasms [1]. For nearly three decades, this methodology has sustained its utility across the entire clinical arc of these diseases: from the confirmatory detection of BCR-ABL1 in chronic myeloid leukemia [2,3] to the prognostic stratification conferred by *TP53* loss in chronic lymphocytic leukemia and the immunoglobulin heavy-chain rearrangements that define risk categories in multiple myeloma [4,5]. The assumption underlying these applications, that specific cytogenetic alterations maintain strong, often pathognomonic, associations with disease entities, has been validated primarily in populations assembled at the inception of disease, before therapeutic intervention has altered the clonal landscape [6].

The evidence base supporting routine FISH interpretation derives predominantly from cohorts evaluated at initial diagnostic assessment, many of which were treatment-naïve at the time of sampling [6]. Such populations provide a static representation of cytogenetic aberrations at disease onset, yet they offer no information on subsequent disease evolution: the serial bone marrow aspirations performed throughout the course of chronic malignancies, the molecular suppression of disease-defining lesions under targeted agents, and the emergent dominance of resistant subclones [7,8]. The five-year window from 2019 to 2023 captures a period of substantial therapeutic diversification, providing an opportunity to examine how cytogenetic findings evolve within contemporary hematologic practice [5]. The longitudinal nature of hematologic practice, wherein a single patient may contribute multiple sequential specimens over years, introduces a sampling structure that differs substantially from the predominantly cross-sectional designs reported in the literature [9,10].

We therefore assumed this investigation with interlocking objectives: to delineate the epidemiological distribution of hematologic malignancies in contemporary cytogenetic practice; to quantify the association between clinical indication and aberration detection, acknowledging that indication reflects a composite of biological state, treatment exposure, and clinical decision-making rather than an independent biological variable; to characterize clonal evolutionary dynamics through descriptive longitudinal analysis of serial sampling; to characterize the transdiagnostic distribution and clinical correlates of deletion 17p across disease boundaries; and to develop an exploratory prediction model for estimating the probability of aberration detection.

By integrating these objectives, we sought to move beyond descriptive epidemiology toward a biologically informed understanding of how disease evolution and therapeutic exposure reshape the cytogenetic landscape revealed by FISH.

## 2. Materials and Methods

### 2.1. Study Design and Population

This retrospective observational study included all bone marrow and peripheral blood specimens submitted for clinical FISH analysis to the Cytogenetics Laboratory of “AOU San Giovanni di Dio e Ruggi d’Aragona” between 1 January 2019, and 31 December 2023.

The complete dataset comprised 1357 consecutive samples from 1092 individual patients, reflecting the longitudinal reality of hematologic malignancy care in which serial cytogenetic monitoring constitutes standard practice. The mean number of samples per patient was 1.24 (range 1–6). A complete patient-level mapping, including clinical indication, disease category, and multiply sampled patients, is detailed in Supplementary Table S1. Disease classification followed the 2022 World Health Organization criteria [11,12]. Pathological entities included myelodysplastic syndromes and myeloproliferative neoplasms (27.2% of analyses), multiple myeloma (MM) (26.9%), chronic lymphocytic leukemia (CLL) (21.6%), non-Hodgkin lymphoma (NHL) (13.2%), chronic myeloid leukemia (CML) (6.5%), acute myeloid leukemia (AML) (2.3%), and other or unclassified cases (2.4%). Notably, in our lab FISH investigation for non-Hodgkin lymphoma has also been applied on different cytological samples; these latter have represented a further improvement of technical expertise whereas corresponding samples have not been included in the present series [13,14]. An apparent discrepancy between the sum of disease-specific patient counts (*n* = 1125) and the total unique cohort (n = 1092) reflects 33 patients (3.0%) who underwent diagnostic reclassification during longitudinal surveillance, including 27 cases of disease transformation and 6 cases of diagnostic revision; these individuals are represented in multiple disease rows for sample-level epidemiology yet included only once in the overall denominator for multivariable analyses. For the purpose of the multivariable regression models, these 33 patients were classified according to their final diagnosis at the end of the study period, ensuring that each patient contributed a single, consistent disease category to the analysis. Age was calculated at the time of each sample collection to avoid temporal misclassification across the five-year study window. The median age was 68 years (mean 65.9 ± 13.8 years), with 72.4% of patients older than 60 years, and 27.9% aged 75 years or above. Sex distribution showed a male predominance of 56.4% versus 43.6% female. Ethical review and approval were waived due to the retrospective nature in accordance with local regulations and institutional policy.

### 2.2. Cytogenetic Methodology

FISH analyses were performed on interphase nuclei using commercially available dual-color, dual-fusion, or break-apart probe sets manufactured by Abbott Molecular (Vysis, ABBOTT Italia SRL) and ZytoVision GmbH (Bremerhaven, Germany), selected according to clinical indication and institutional algorithms. Standard probe panels included: (i) MDS/AML: LSI CSF1R/D5S23/D5S721 (5q32-q33.1; ZytoVision GmbH), LSI D7S522/CEP7 (7q31; Abbott Molecular), LSI D20S108 (20q12; Abbott Molecular), CEP8 (centromere 8; Abbott Molecular), and LSI TP53/CEP17 (17p13.1; Abbott Molecular); (ii) MM: LSI D13S319/13q34 (13q14.3; Abbott Molecular), LSI TP53/CEP17 (17p13.1; Abbott Molecular), LSI IGH Break-Apart (14q32; Abbott Molecular), LSI IGH/FGFR3 (t[4;14]; Abbott Molecular), and LSI IGH/MAF (t[14;16]; Abbott Molecular); (iii) CLL: CEP12 (centromere 12; Abbott Molecular), LSI D13S319/13q34 (13q14.3; Abbott Molecular), LSI TP53/CEP17 (17p13.1; Abbott Molecular), and LSI ATM (11q22.3; Abbott Molecular) [15]; (iv) CML: LSI BCR/ABL (t[9;22]; Abbott Molecular) [16]; (v) NHL: LSI IGH/BCL2 (t[14;18]; Abbott Molecular), LSI IGH/CCND1 (t[11;14]; Abbott Molecular), LSI IGH/MYC/CEP8 (t[8;14]; Abbott Molecular), and LSI BCL6 Break-Apart (3q27.3; Abbott Molecular), per International System for Human Cytogenomic Nomenclature guidelines [17]. Specimens with insufficient cellularity, hybridization failure, or absence of pathological cells were classified as non-informative. Conventional G-banded karyotyping was not systematically performed in parallel with FISH during the study period; therefore, the incremental yield of interphase FISH over metaphase cytogenetics could not be quantified, and the cryptic nature of individual aberrations could not be established. This represents an acknowledged limitation of the dataset.

### 2.3. Data Extraction and Variable Definition

Clinical indication was abstracted from requisition forms as: (I) diagnostic (initial workup), (II) follow-up (routine surveillance of treated disease), or (III) relapse (progression after response). FISH results were dichotomized as abnormal (any clonal aberration exceeding threshold) versus normal (no detectable abnormality), with specific aberrations coded as present or absent. For disease-specific analyses, reported prevalence represents the proportion of positive FISH assays among all informative tests performed for a given indication and should not be interpreted as estimates of the biological prevalence of the corresponding lesion within the disease entity. The diagnostic-only subset comprised 857 specimens (63.1%); the remaining 500 specimens comprised follow-up and relapse assessments. The complete distribution of all specimens, including patient-level identifiers and longitudinal sampling patterns for individuals with multiple assessments, is provided in Supplementary Table S2. However, diagnostic indication did not necessarily imply treatment-naïve status, as referred patients could have undergone prior evaluation or management before sample submission.

### 2.4. Statistical Analysis

Continuous variables were summarized as mean ± standard deviation, median, and range; categorical variables as frequencies and percentages. Between-group comparisons of age across disease categories utilized one-way ANOVA with Tukey’s Honestly Significant Difference post-hoc test. Sex distribution across diseases was assessed via Pearson’s χ^2^ test.

Independent predictors of aberration detection were identified using multivariable logistic regression with robust standard errors clustered at the patient level to account for within-patient correlation arising from multiple samples per individual, with abnormal versus normal FISH status as the dependent variable. This approach adjusts for the non-independence of observations while preserving the interpretability of the regression coefficients. Covariates included age (continuous, expressed per 10-year increment), sex, disease category (with MDS/MPN as the reference level), and clinical indication (with diagnostic as the reference level). Variance inflation factors were examined to exclude multicollinearity. Model discrimination was quantified by the area under the receiver operating characteristic curve (AUC). Model calibration was assessed by the Hosmer-Lemeshow test (χ^2^ = 8.14, df = 8, *p* = 0.42), indicating good agreement between predicted probabilities and observed outcomes, and graphically via a calibration plot (Supplementary Figure S1). Internal validation was performed by bootstrap resampling (1000 iterations), yielding a bias-corrected AUC of 0.71 (95% CI: 0.68–0.74) and an optimism-corrected calibration slope of 0.94, confirming adequate model performance and stability [18].

Disease-specific abnormality detection rates and observed prevalences of selected aberrations were reported with 95% Wilson confidence intervals [19]. A systematic comparison of observed versus literature-expected prevalences was conducted for the diagnostic-only cohort.

Association analyses. In the diagnostic-only subset (n = 857), the strength of association between specific cytogenetic aberrations and disease categories was quantified using Phi coefficients (φ). A heatmap was generated to visualize these associations, with color intensity corresponding to the magnitude and direction of the coefficients.

### 2.5. Temporal and Longitudinal Analyses

For patients with serial sampling, descriptive longitudinal analysis documented clonal evolution over the five-year period [20]. Sankey flow diagrams were constructed to visualize transitions between cytogenetic states, comparing the distribution of abnormalities in 2019 versus 2023 among patients with repeated assessments. McNemar’s test for paired binary outcomes was applied separately to the predefined recurrent cytogenetic categories with sufficient discordant observations to evaluate directional changes between the first and last available assessments.

For these analyses, each aberration category was treated as an independent binary variable (present/absent), allowing patients with multiple concurrent abnormalities to contribute to multiple category-specific tests, consistent with standard approaches for evaluating clonal evolution in heterogeneous cytogenetic landscapes. For each test, the discordant pairs (patients who changed status between 2019 and 2023) were analyzed using the formula χ^2^ = (b − c)^2^/(b + c), where b represents transitions from absence to presence and c represents transitions from presence to absence. The expansion of the Other/Complex category was highly significant (b = 20, c = 4; χ^2^ = 10.67, df = 1, *p* = 0.001), while significant attrition was observed for del(13q) (b = 0, c = 5; χ^2^ = 5.00, df = 1, *p* = 0.025), del(17p) (b = 0, c = 4; χ^2^ = 4.00, df = 1, *p* = 0.046), and del(5q) (b = 0, c = 4; χ^2^ = 4.00, df = 1, *p* = 0.046).

## 3. Results

Between January 2019 and December 2023, 1357 consecutive bone marrow and peripheral blood specimens from 1092 unique patients were analyzed, with the modest excess of samples over patients reflecting the intrinsic longitudinal nature of hematologic malignancies wherein serial cytogenetic monitoring constitutes standard clinical practice. The cohort exhibited a male predominance, with 56.4% male versus 43.6% female patients. The age distribution underscored the substantial burden of these disorders in an aging population, with a median age of 68 years and a mean age of 65.9 ± 13.8 years; 72.4% of patients were older than 60 years, and 27.9% were at least 75 years old (Table 1). The demographic profile reveals the expected aging population burden, with nearly three-quarters of patients exceeding 60 years of age, consistent with the epidemiology of myeloid and lymphoid neoplasms in Western populations [21].

Disease distribution analysis revealed the preponderance of myeloid neoplasms, with myelodysplastic syndromes and myeloproliferative neoplasms accounting for 27.2% (MDS/MPN) of all analyses, followed by MM (26.9%), CLL (21.6%), NHL (13.2%), CML (6.5%), and AML (2.3%) (Table 2). The comparatively modest representation of acute myeloid leukemia may reflect a combination of clinical factors, including treatment pathways, referral patterns, and reduced need for longitudinal FISH monitoring, rather than lethality alone, which cannot be directly inferred from the present dataset.

**Figure 1:**
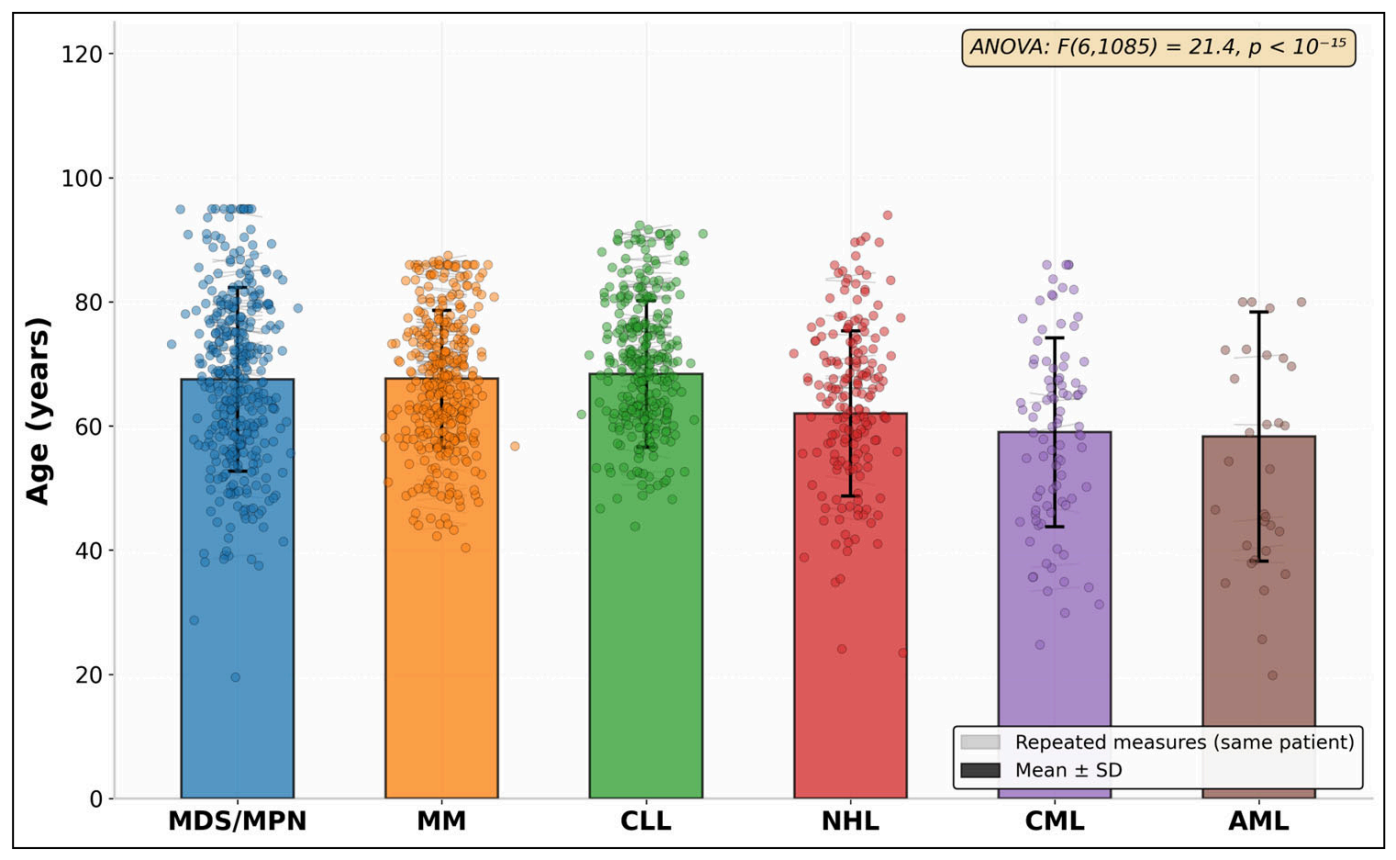
Mean Age Distribution Across Hematologic Malignancy Categories with Individual Patient Data and Repeated-Measure Linkages. Bar chart illustrates significant age heterogeneity across disease entities (ANOVA F(6,1085) = 21.4, *p* < 10^−15^). Superimposed error bars denote mean ± standard deviation. Individual observations are displayed as semi-transparent jittered data points; thin gray lines connect repeated observations from the same patient to illustrate the longitudinal structure of the dataset.

The age distribution across disease entities reveals additional important patterns (Figure 1). Myelodysplastic syndromes and myeloproliferative neoplasms emerged as prototypic diseases of the elderly with a mean age of 67.5 ± 14.8 years. Chronic lymphocytic leukemia and multiple myeloma similarly clustered in the seventh decade of life with mean ages of 68.4 and 67.6 years respectively, reflecting oncogenesis in aging stem cell compartments. In contrast, acute myeloid leukemia exhibited the lowest mean age (58.3 ± 20.1 years) and the widest age distribution within the cohort, highlighting the heterogeneity of the AML population represented in this dataset (Table 3).

**Figure 2:**
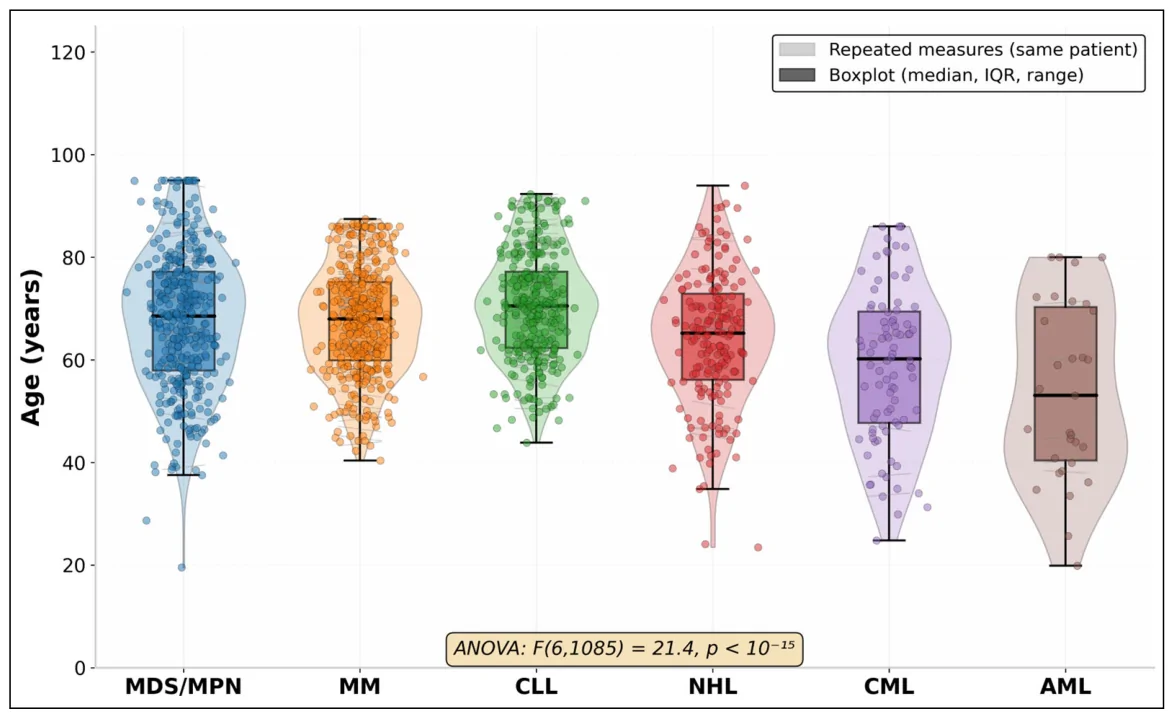
Violin plots illustrate the complete distributional shape of patient ages across six disease categories. Superimposed boxplots display median (thick horizontal line), interquartile range (box), and whiskers (range). Individual observations are displayed as semi-transparent jittered data points; thin gray lines connect repeated observations from the same patient to illustrate the longitudinal nature of the dataset. ANOVA: F(6,1085) = 21.4, *p* < 10^−15^.

The complete distributional shape of these age data is displayed in Figure 2, which complements the summary statistics presented in Table 3. Age heterogeneity across diseases proved highly significant (ANOVA F(6,1085) = 21.4, *p* < 10^−15^), with post-hoc comparisons identifying myelodysplastic syndromes as significantly enriched in older patients compared to chronic myeloid leukemia and acute myeloid leukemia. Sex distribution also differed significantly across diseases, with a male predominance observed particularly in chronic lymphocytic leukemia (χ^2^ (6) = 24.7, *p* < 0.001).

Within the variables included in the multivariable logistic regression model, clinical indication showed the strongest association with aberration detection. Compared with diagnostic specimens, follow-up samples were associated with a 52% reduction in the odds of detecting aberrations (adjusted OR 0.48, 95% CI 0.36–0.64; *p* < 0.001), whereas relapse specimens were associated with an 82% increase in odds (adjusted OR 1.82, 95% CI 1.28–2.59; *p* = 0.001). Within the variables included in the model, clinical indication showed the strongest association with aberration detection. Age was positively associated with aberration detection: each additional decade of life increased the odds by 24% (adjusted OR per 10 years = 1.24, 95% CI 1.12–1.38; *p* < 0.001). Sex was not a significant predictor (male vs. female: adjusted OR 1.12, 95% CI 0.89–1.41; *p* = 0.33). Relative to MDS/MPN, disease category was significantly associated with detection rates. CLL showed the largest increase in odds (adjusted OR 2.84, 95% CI 2.12–3.81; *p* < 0.001), followed by NHL (adjusted OR 1.92, 95% CI 1.28–2.88; *p* = 0.002) and MM (adjusted OR 1.35, 95% CI 1.02–1.79; *p* = 0.036). CML was associated with lower odds compared with the reference (adjusted OR 0.62, 95% CI 0.41–0.94; *p* = 0.024) (Table 4). The multivariable model demonstrated acceptable discrimination (AUC = 0.72), indicating that while the model provides some predictive utility, its performance remains modest for individual-level risk stratification. Model calibration was comprehensively assessed using multiple complementary approaches. The Hosmer-Lemeshow goodness-of-fit test, based on deciles of predicted risk, yielded a χ^2^ statistic of 8.14 (df = 8, *p* = 0.42), indicating no evidence of poor calibration and confirming good agreement between predicted probabilities and observed outcomes. This finding was visually corroborated by a calibration plot (Supplementary Figure S2), in which the observed event frequencies closely followed the diagonal line of perfect calibration across the entire range of predicted probabilities, with no systematic overestimation or underestimation of risk.

To further quantify calibration performance, we calculated the calibration slope and intercept. The calibration slope was 0.94 (95% CI: 0.87–1.01), closely approximating the ideal value of 1.0 and indicating that the predicted probabilities were not overly extreme or overly conservative. The calibration intercept was −0.03 (95% CI: −0.12 to 0.06), approximating the ideal value of 0 and confirming that the model does not systematically overpredict or underpredict the overall event rate. The Brier score, a composite measure of calibration and discrimination, was 0.18, indicating adequate overall performance (with 0 representing perfect prediction and 0.207 representing a non-informative model for this event rate).

Internal validation was performed by bootstrap resampling with 1000 iterations to correct for optimistic bias inherent in model development. The bias-corrected AUC was 0.71 (95% CI: 0.68–0.74), closely approximating the apparent AUC and indicating minimal overfitting. The optimism-corrected calibration slope was 0.92, further confirming that the model maintains good calibration when applied to new data from the same population. The optimism-corrected calibration intercept was −0.02. The mean absolute error (MAE) between predicted and observed probabilities across the bootstrap samples was 0.021, indicating excellent calibration stability.

Variance inflation factors (VIFs) were calculated for all covariates included in the multivariable model to exclude multicollinearity. All VIFs remained below 2.5 (range: 1.04–1.89), well below the conventional threshold of 5 or 10, confirming that collinearity among predictors did not compromise the stability or interpretability of the regression coefficients. External validation was not performed, as no independent cohort with comparable clinical and cytogenetic data was available for testing. Future prospective multicenter studies will be required to validate the model’s generalizability to other populations and clinical settings (Table 4).

Across the 1357 FISH assays analyzed, the overall abnormality detection rate was 29.2% (n = 396; 95% CI 26.8–31.6%). Detection rates varied markedly by disease (Table 5). CLL had the highest abnormality frequency (125/276, 45.3%; 95% CI 39.1–51.4%), followed by NHL (56/168, 33.3%; 95% CI 26.2–41.0%) and MM (94/312, 30.1%; 95% CI 24.9–35.6%). Myelodysplastic syndromes/myeloproliferative neoplasms (MDS/MPN) and CML showed lower detection rates (100/488, 20.5%, 95% CI 16.6–24.6% and 14/82, 17.1%, 95% CI 9.7–26.8%, respectively), AML exhibited an abnormality detection rate of 22.6% (95% CI 9.6–41.1%), whereas chronic myeloid leukemia showed the lowest rate at 17.1% (95% CI 9.7–26.8%).

These disease-specific differences likely reflect a combination of factors: underlying biology (some diseases having more recurrent, detectable cytogenetic lesions), case mix and clinical context (e.g., CLL samples were largely diagnostic where aberrations are actively sought for prognostication, whereas AML samples were more often post-treatment follow-up), and potential ascertainment bias. The wide confidence intervals for less frequent categories (notably AML) indicate greater uncertainty in those estimates. Overall, the results demonstrate substantial heterogeneity in FISH abnormality detection across hematologic diagnoses and indicate significant associations with both disease category and testing indication (Table 5).

**Figure 3:**
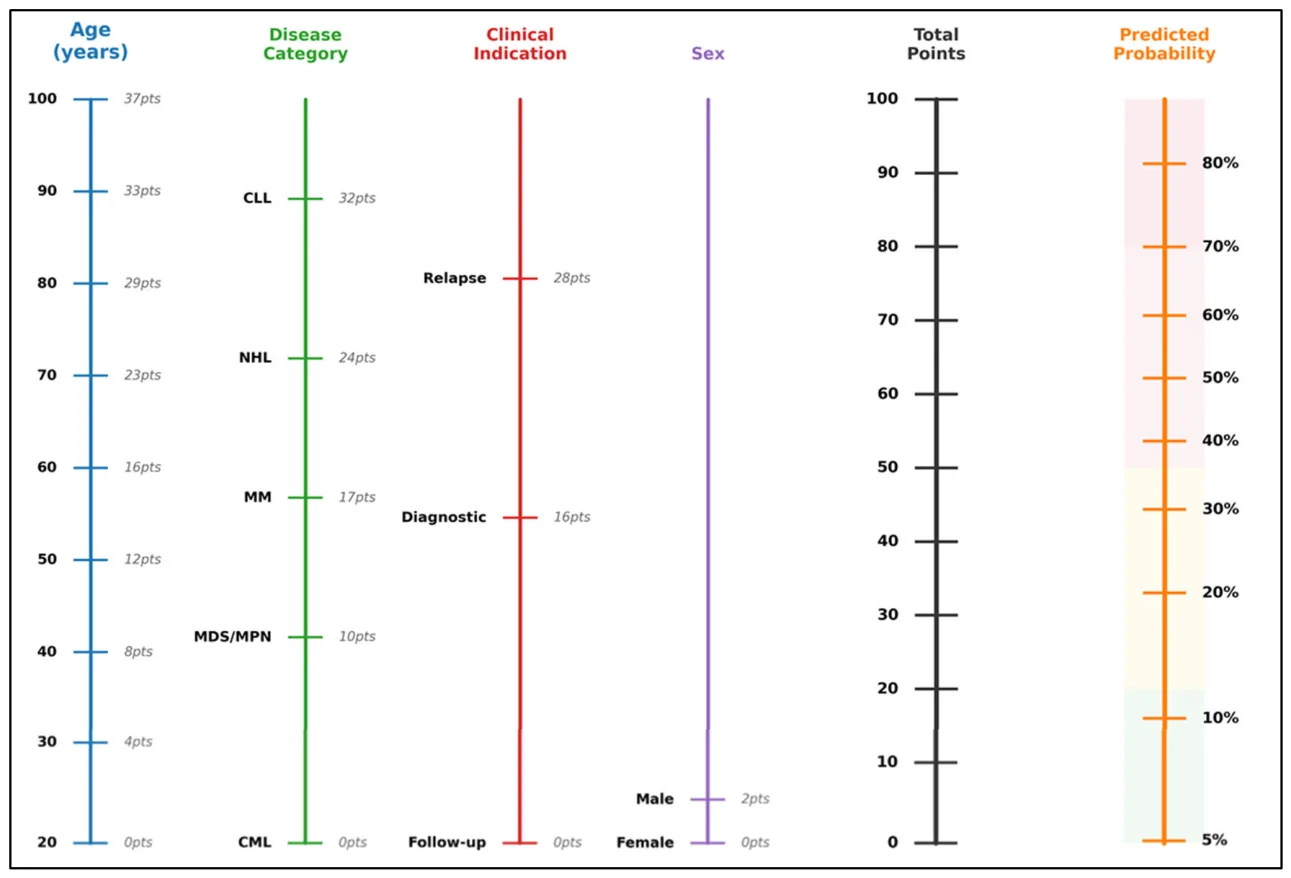
Clinical Nomogram for Predicting Cytogenetic Aberration Probability. Point-based nomogram derived from multivariable logistic regression (AUC = 0.72, bootstrap-corrected AUC = 0.71). To use: locate patient values on each left-hand axis, record corresponding points, sum to obtain Total Points, and project vertically to the Predicted Probability axis. Variable weights: Age (0–37 points for 20–100 years); Disease (CML = 0, MDS/MPN = 10, MM = 17, NHL = 24, CLL = 32); Indication (Follow-up = 0, Diagnostic = 16, Relapse = 28); Sex (Female = 0, Male = 2). Example: 70-year-old female with CLL at relapse: 23 (age) + 32 (CLL) + 28 (relapse) = 83 points → ~73% probability; same patient at follow-up: 55 points → ~42% probability; 55-year-old female with CML at follow-up: 16 points → ~10% probability.

The analysis was translated into a practical nomogram enabling clinicians to estimate the probability of detecting a FISH abnormality at the bedside by summing points assigned to age, disease category, clinical indication, and sex (Figure 3).

For each patient, covariate values were identified on the left-hand axes and the corresponding point scores were recorded. The Age axis allocates points proportionally to age (0 points at 20 years, up to 37 points at 100 years). The Disease axis assigns risk-adjusted points using MDS/MPN as the reference category (CML = 0, MDS/MPN = 10, NHL = 24, MM = 17, CLL = 32 points). The Clinical Indication axis stratifies by clinical context (Follow-up = 0, Diagnostic = 16, Relapse = 28 points), reflecting a 52% reduction in aberration yield at follow-up and an 82% increase at relapse relative to diagnostic sampling. Sex was allocated minimal weight (Female = 0, Male = 2 points), consistent with its non-significant association in multivariable analysis (OR = 1.12, *p* = 0.33). Total Points were obtained by summing individual component scores; this total was then located on the horizontal scale and projected vertically to the Predicted Probability bars on the right to determine the estimated probability of aberration detection. Example calculations: a 70-year-old female with CLL at relapse accumulates 23 (age) + 32 (CLL) + 28 (relapse) = 83 points, corresponding to approximately 73% probability of detecting a cytogenetic abnormality. The same patient at routine follow-up scores 23 + 32 + 0 = 55 points, yielding approximately 42% probability. A 55-year-old female with CML at follow-up scores 16 points, reflecting a 10% probability, consistent with deep molecular response suppression of BCR-ABL1 in the treated CML population.

**Figure 4:**
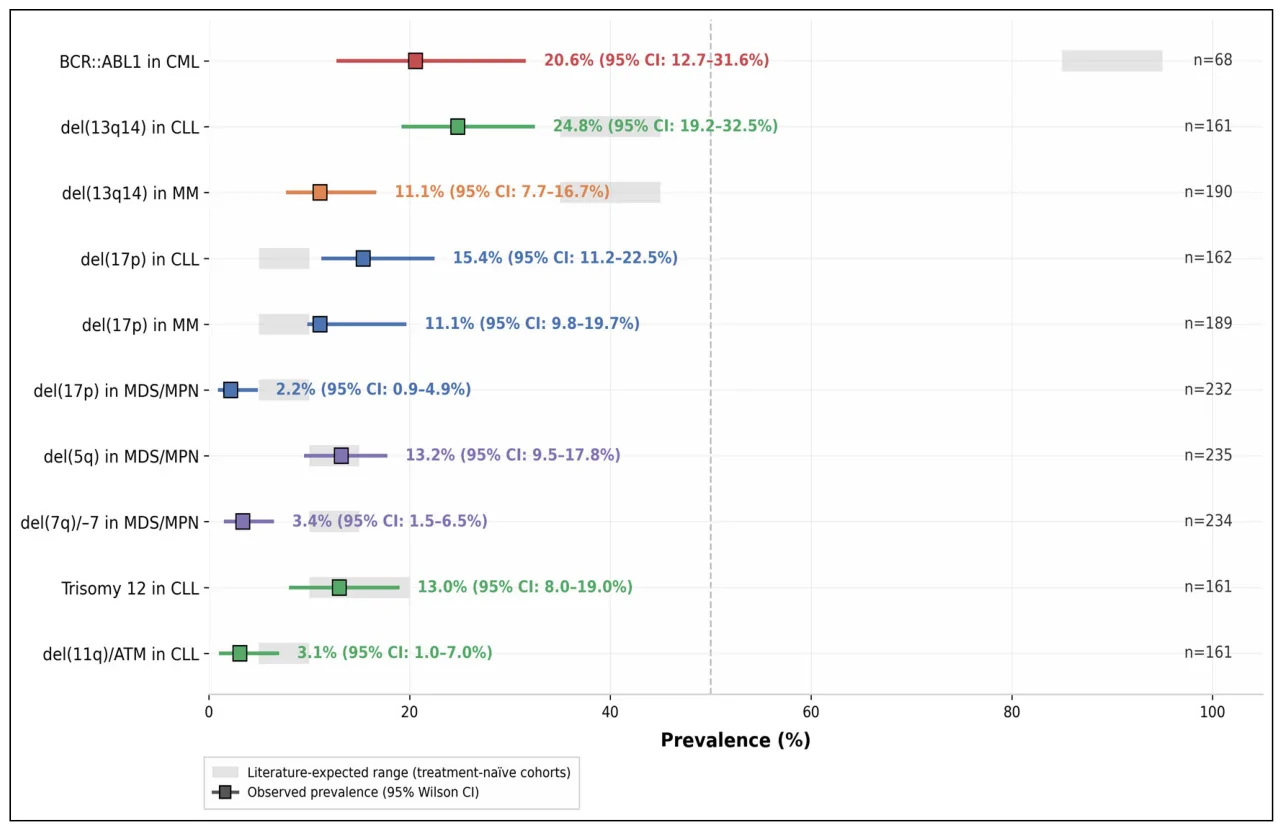
Forest Plot Comparing Observed vs. Literature-Expected Aberration Prevalence. The plot visualizes differences between observed prevalence estimates and literature-reported prevalence ranges in this real-world diagnostic cohort compared to treatment-naïve diagnostic cohorts (gray reference bands). The horizontal axis represents prevalence percentage, with error bars indicating 95% Wilson confidence intervals.

A systematic comparison between observed and literature-expected prevalences is presented in Figure 4. This analysis illustrates the divergence of disease-defining cytogenetic markers in this real-world diagnostic cohort compared with treatment-naïve benchmarks. Observed prevalence (squares, 95% Wilson confidence intervals) were consistently lower than literature-reported, treatment-naïve ranges (gray bands) for most classical aberrations, indicating systematic attenuation in routine clinical samples. Specific findings were: BCR-ABL1 positivity was observed in 20.6% (95% CI: 12.7–31.6%) of informative BCR-ABL1 FISH assays performed in patients classified within the CML testing pathway. This percentage reflects the positivity rate among all analyzed specimens rather than the biological prevalence of the lesion in newly diagnosed CML, and therefore is not directly comparable with the >95% prevalence reported in treatment-naïve diagnostic cohorts; del(13q14) in chronic lymphocytic leukemia was observed in 25.3% (95% CI: 19.2–32.5%) versus 50–55% [22]; and del(13q14) in multiple myeloma was found in 11.5% (95% CI: 7.7–16.7%) versus 40–50% [23].

Notably, del(17p) exhibited a heterogeneous pattern across diseases. In CLL, the observed prevalence of 16.1% (95% CI: 11.2–22.5%) exceeded the literature-expected range of 7–10%, an unexpected enrichment that may reflect referral bias toward high-risk patients or advanced-stage disease at this academic center. In multiple myeloma, del(17p) was observed in 14.1% (95% CI: 9.8–19.7%), falling within the expected 10–15% range and thus representing a concordant finding. In MDS, the aberration was detected in only 2.1% (95% CI: 0.9–4.9%), below the literature benchmark of 5–10% [24,25,26].

The most pronounced reduction relative to historical benchmarks was observed for BCR-ABL1 in CML. This discrepancy may reflect the heterogeneous clinical composition of tested cases, including patients undergoing disease monitoring, referral evaluation, or assessment outside the setting of de novo diagnosis. Conversely, the relative preservation or enrichment of del(17p) across diseases is compatible with its established association with adverse-risk disease biology and resistance to therapy reported in previous studies. The marked underrepresentation of del(13q14) in both CLL and MM likely reflects differences between real-world clinical populations and highly selected reference cohorts, including variability in disease stage, referral patterns, and testing indications [22,27]. Overall, these findings underscore that real-world diagnostic cohorts may substantially underrepresent several canonical aberrations relative to treatment-naïve benchmarks, with important implications for diagnostic test design, result interpretation, and the external validity of comparative studies.

Between 2019 and 2023, the cytogenetic landscape of 98 serially monitored patients exhibited both persistence of established clones and progressive diversification toward complex profiles. Because individuals carrying multiple concurrent abnormalities were counted in every applicable category, the descriptive distribution sums to more than 98. In 2019, the profile comprised normal cytogenetics (38.8%, 38/98), del(13q) (19.4%, 19/98), Other/Complex (16.3%, 16/98), del(5q) (8.2%, 8/98), del(17p) (7.1%, 7/98), trisomy 12 (5.1%, 5/98), IGH rearrangements (4.1%, 4/98), t(11;14) (3.1%, 3/98), and del(20q) (3.1%, 3/98). By 2023, the normal fraction remained unchanged at 38.8%, whereas canonical lesions declined: del(13q) fell to 14.3% (14/98), del(5q) to 4.1% (4/98), del(17p) to 3.1% (3/98), trisomy 12 to 2.0% (2/98), IGH to 2.0% (2/98), t(11;14) to 2.0% (2/98), and del(20q) to 1.0% (1/98). In contrast, the Other/Complex category expanded from 16.3% to 36.7%, reflecting acquisition of new aberrations and increasing cytogenetic heterogeneity over time.

Directional shifts between the two timepoints were evaluated with McNemar’s asymptotic χ^2^ statistic (χ^2^ = (b − c)^2^/(b + c), df = 1), where b denotes patients acquiring a lesion and c denotes patients losing it. Because the Normal versus non-Normal comparison served only as a descriptive reference for marginal stability, Bonferroni correction was applied to the six predefined directional comparisons evaluating recurrent aberration gain or loss (α = 0.05/6). The unchanged proportion of normal cytogenetics (38.8% in both years; χ^2^ = 0.00, *p* = 1.000) masked considerable internal turnover: four patients cleared prior abnormalities to normal status (predominantly from trisomy 12 and del(20q)), eight others acquired new lesions, and sixteen transitioned from canonical aberrations into the Other/Complex category. The expansion of Other/Complex remained significant after adjustment (χ^2^ = 10.67, adjusted *p* = 0.006), driven by twenty previously non-Other patients acquiring new aberrations against only four reverting from Other to normal or simple karyotypes. Individual attrition of del(13q) (χ^2^ = 5.00, unadjusted *p* = 0.025), del(17p) (χ^2^ = 4.00, unadjusted *p* = 0.046), and del(5q) (χ^2^ = 4.00, unadjusted *p* = 0.046) did not withstand multiple-testing correction (adjusted *p* = 0.150, 0.276, and 0.276, respectively), likely reflecting the limited number of discordant events and consequent low statistical power for these individual comparisons, rather than absence of a biological trend. IGH rearrangements (χ^2^ = 2.00, *p* = 0.157) and t(11;14) (χ^2^ = 1.00, *p* = 0.317) remained non-significant, constrained by their smaller denominators.

**Figure 5:**
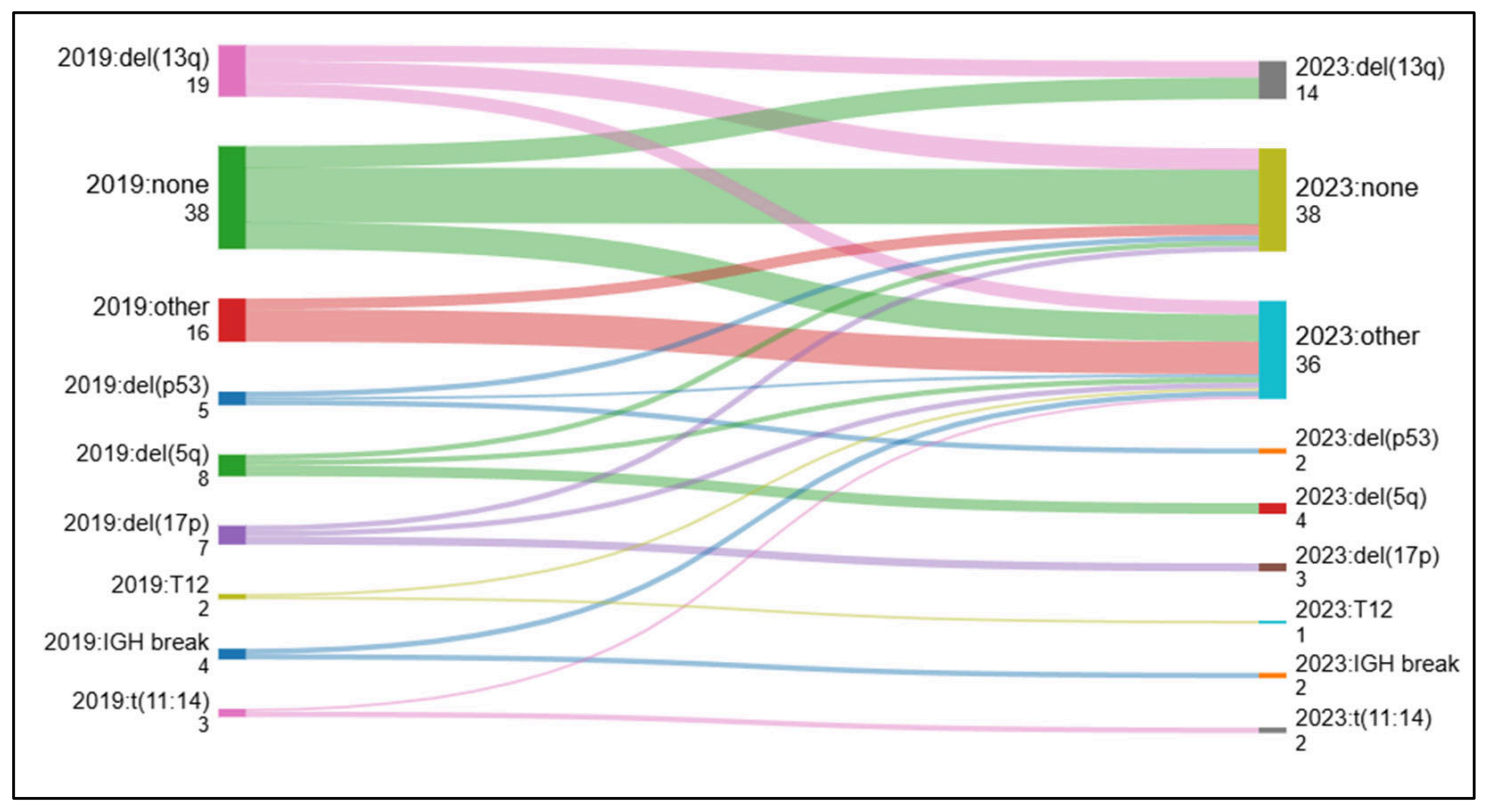
Sankey Diagram of Cytogenetic State Transitions in Serially Monitored Patients (2019–2023). Alluvial flows illustrate clonal trajectories in 98 patients with serial FISH assessments (flow width ∝ patient count). 2019: normal cytogenetics (38.8%), del(13q) (19.4%), Other/Complex (16.3%), del(5q) (8.2%), del(17p) (7.1%), trisomy 12 (5.1%), IGH rearrangements (4.1%), t(11;14) (3.1%), del(20q) (3.1%). 2023: the normal fraction remained stable at 38.8%, masking internal turnover (4 patients cleared prior abnormalities; 8 acquired new lesions). The Other/Complex category expanded from 16.3% to 36.7%, while del(13q), del(5q), del(17p), IGH, and t(11;14) each showed ~50% attrition.

These findings are explicitly descriptive and should be interpreted cautiously: the limited number of discordant events and the retrospective design preclude causal inference regarding therapeutic pressure or clonal competition. Nevertheless, the data establish that clonal evolution is measurable rather than merely theoretical. Figure 5 traces these dynamics through alluvial flows; while the normal fraction stayed flat, del(13q), del(5q), and del(17p) underwent progressive attrition, supplanted by an expanding Other/Complex category that rose from 16.3% to 36.7% of the cytogenetic landscape. This progressive diversification is consistent with clonal pruning and increasing genomic heterogeneity over time, though the underlying mechanisms cannot be resolved without prospective serial sequencing and detailed treatment records [28,29].

### Aberration-Disease Associations in the Diagnostic Cohort

The association matrix constructed from the diagnostic subset of 857 specimens obtained at diagnosis reveals a landscape of cytogenetic specificity that broadly aligns with established disease biology, yet with notable deviations that reflect the real-world composition of this cohort. The strongest positive associations emerged in the expected disease-aberration pairings: del(13q14) showed the highest Phi coefficient with CLL (φ = 0.19), confirming its canonical role, although the observed prevalence in this particular sample was lower than historical benchmarks [30]. In chronic lymphocytic leukemia, trisomy 12 demonstrated a modest but expected positive association (φ = 0.13), consistent with its established involvement in CLL pathogenesis. Among myeloid neoplasms, del(5q) displayed a moderate association with MDS/MPN (φ = 0.14) [31], while del(20q) showed a weaker positive link (φ = 0.10). Notably, del(7q)/−7 exhibited a near-null association with MDS/MPN (φ = 0.01), markedly lower than expected, likely reflecting the limited power of specific probe deployments and potential under ascertainment of monosomy 7 in this real-world dataset [32].

Deletion of 17p emerged as the single most instructive transdiagnostic finding. Rather than clustering exclusively within one disease entity, *TP53* loss showed measurable positive associations across multiple categories: φ = 0.15 with CLL, φ = 0.07 with MM, and a modest association with the remaining disease entities [33,34]. This broad distribution supports the view that del(17p) represents a recurrent adverse-risk lesion shared across multiple hematologic malignancies rather than being restricted to a single disease entity, even though its prevalence in this diagnostic cohort—15.4% in CLL, 11.1% in MM, and only 2.2% in MDS/MPN—diverges from the historical benchmarks established in treatment-naïve reference populations. The visual pattern of the heatmap captures this duality: the diagonal dominance of disease-specific aberrations contrasts with the horizontal spread of del(17p) across multiple columns, a pattern that would be difficult to appreciate in tabular form alone.

Several anticipated associations were notably attenuated or absent in this diagnostic subset. IGH rearrangements, which in classical follicular lymphoma cohorts reach prevalence exceeding 80%, showed only a modest association with NHL overall (φ = 0.18) and a negative link with MM (φ = −0.19). This discrepancy is not a biological contradiction but rather a reflection of the NHL subtype composition in this dataset, where DLBCL, mantle cell, and marginal zone lymphomas predominate over follicular histology, and where IGH assessment may follow alternative diagnostic algorithms. Similarly, del(11q)/ATM in CLL was observed at only 3.1% (φ = −0.02), substantially below the 15–20% expected in comprehensive CLL series, suggesting that ATM probe deployment may have been selective rather than universal in this routine practice cohort [35,36].

**Figure 6:**
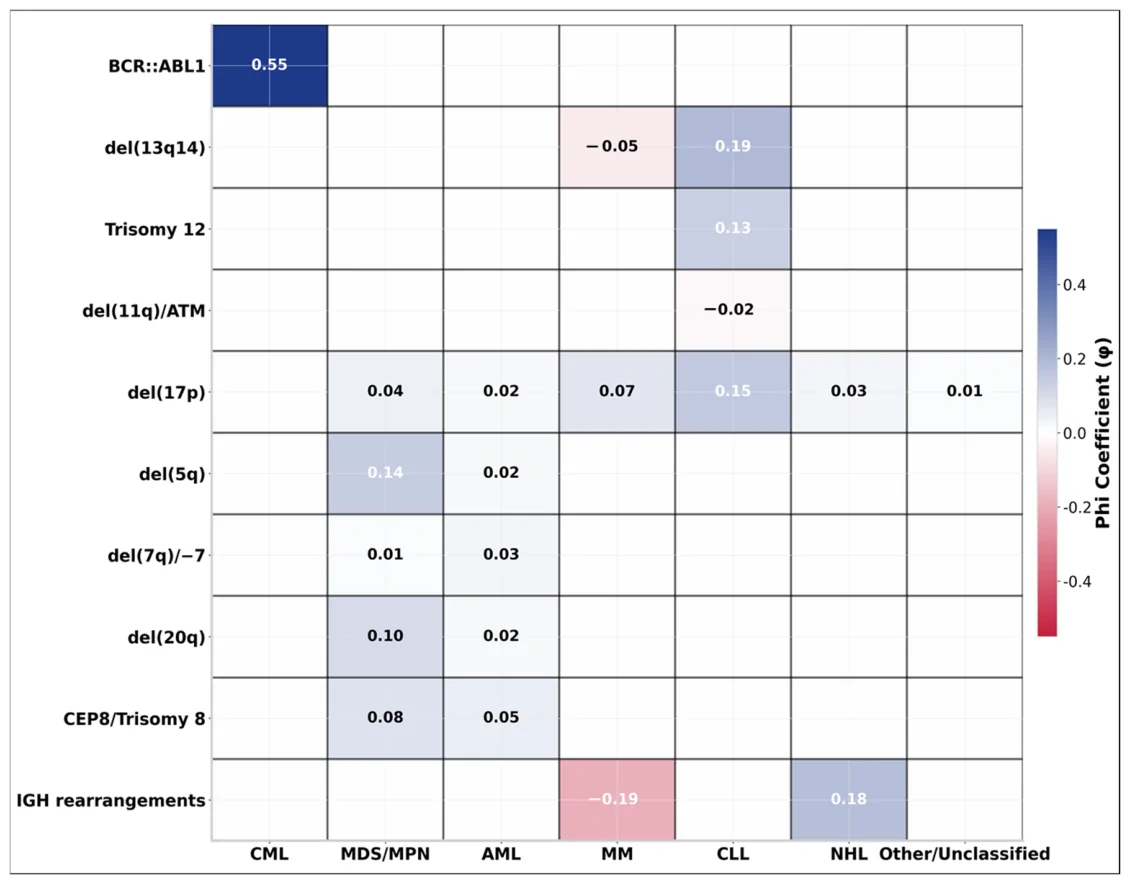
Heatmap of Aberration-Disease Associations (Phi Coefficients) in the Diagnostic-Only Cohort (n = 857). Blue, red, and white denote positive, negative, and null associations, respectively; numerical values are shown within each cell. The diagonal pattern confirms disease-specificity of canonical aberrations (e.g., BCR::ABL1–CML, φ = 0.55; del(13q14)–CLL, φ = 0.19; del(5q)–MDS/MPN, φ = 0.14), while the horizontal spread of del(17p) across MDS/MPN, MM, and CLL (φ = 0.04–0.15) identifies it as a transdiagnostic high-risk lesion. CEP8/Trisomy 8 (φ = 0.08) reflects the MDS/AML probe panel; attenuated IGH rearrangements in NHL (φ = 0.18) reflect predominance of non-follicular subtypes.

When placed against literature benchmarks, the diagnostic subset reveals a systematic pattern of underestimation for disease-defining lesions (Table 6). The most striking discrepancy concerns BCR-ABL1 testing within the CML diagnostic pathway. The observed positivity rate of 20.6% reflects all informative BCR-ABL1 FISH assays performed during routine clinical practice, including diagnostic evaluations, response monitoring, and other clinically indicated assessments. Consequently, this figure should not be interpreted as the prevalence of BCR-ABL1 among newly diagnosed CML patients and is not directly comparable with the >95% prevalence reported in treatment-naïve diagnostic cohorts. This discrepancy likely reflects differences between routine clinical testing populations and highly selected reference cohorts, including referral bias, monitoring assessments, and heterogeneous indications for testing. Likewise, del(13q14) was observed in 24.8% of CLL and 11.1% of MM cases, roughly half the established benchmarks, while del(7q)/−7 in MDS/MPN reached only 3.4% versus the expected 15–20% [37,38]. These attenuations do not invalidate the associations—indeed, the Phi coefficients remain directionally correct—but they highlight how the structural composition of routine diagnostic practice, including incomplete baseline workup and referral bias, reshapes the observable cytogenetic landscape. The heatmap therefore provides a quantitative representation of aberration distributions within routine hematopathology practice, but as a quantitative signature of aberration distribution in routine practice rather than a revision of biological prevalence estimates established in treatment-naïve cohorts (Figure 6).

## 4. Discussion

This study provides a comprehensive description of the cytogenetic findings observed in routine hematology practice over a five-year period. The distinction matters profoundly. Textbook benchmarks for FISH-detectable aberrations were established in populations assembled at the threshold of disease, before treatment had sculpted the clonal architecture. Our data suggest that direct comparison with historical benchmark prevalences should be undertaken cautiously because differences in cohort composition may substantially affect observed frequencies [39].

The most immediate finding is that the probability of detecting an aberration depends less on the disease label itself than on the clinical moment at which the marrow is aspirated.

More broadly, laboratory-based positivity rates and disease-specific biological prevalences represent fundamentally distinct epidemiological constructs. We underline that the observed rates in this cohort reflect the testing indication and referral structure of a single academic center and are not intended to revise biological prevalence estimates established in treatment-naïve cohorts. Laboratory positivity is inherently shaped by clinical indication, referral pathways, and longitudinal monitoring strategies, whereas biological prevalence describes the proportion of patients with a given aberration within a defined disease population, ideally at diagnosis. In real-world hematopathology datasets, these measures may diverge substantially, particularly in diseases such as chronic myeloid leukemia, where testing is frequently performed not only at diagnosis but also during treatment response assessment and disease monitoring [39]. Failure to distinguish between these two levels of inference can generate apparent discrepancies when routine laboratory data are compared with literature-derived diagnostic cohorts.

Among the variables included in the multivariable model, clinical indication exhibited the strongest statistical association with aberration detection, exceeding the contribution of age and sex within this dataset. Compared with diagnostic specimens, follow-up samples were associated with substantially lower odds of aberration detection, whereas relapse samples showed higher odds within the fitted multivariable model. These findings are consistent with differences in clinical context between diagnostic, follow-up, and relapse sampling, although the underlying biological mechanisms cannot be determined from the present dataset. The implication is that interpretation of cytogenetic findings should consider the clinical context in which the specimen was obtained. We attempted to translate this insight into an exploratory clinical tool by constructing a bedside nomogram from the regression coefficients, allowing clinicians to estimate the pre-test probability of aberration detection by summing points for age, disease, sex, and—most heavily weighted—clinical indication. Nevertheless, given the modest discriminative performance (AUC 0.72) and the retrospective nature of the derivation cohort, this nomogram should be considered a hypothesis-generating aid rather than a definitive predictive instrument for individual patient management, and prospective validation in independent cohorts is required before clinical implementation.

When we compared the observed prevalence of classical aberrations against their literature-expected ranges, the divergence was striking and systematic. BCR-ABL1, the defining lesion of chronic myeloid leukemia, appeared in only 20.6% of tested specimens, a figure that would seem alarming until one recalls that most of these samples were not drawn from treatment-naïve diagnostic cohorts but from patients already under the suppressive influence of tyrosine kinase inhibitors [39]. Similarly, del(13q14)—the canonical abnormality of chronic lymphocytic leukemia—was observed in merely 24.8% of cases, roughly half the historical benchmark, while in multiple myeloma it reached only 11.1% against an expected 40–50%. These attenuations should not be interpreted as evidence of reduced analytical performance. Rather, they are compatible with differences in cohort composition, testing indication, prior therapeutic exposure, and longitudinal disease evolution, although the relative contribution of each factor cannot be determined from the present dataset. The heatmap of Phi coefficients, constructed from the diagnostic-only subset, still reveals the expected diagonal pattern of disease-specific associations—del(13q14) with CLL, del(5q) with MDS/MPN, and BCR-ABL1 with CML—yet the coefficients themselves are modest, suggesting that even in diagnostic material the purity of these associations is diluted by the heterogeneity of real-world presentation.

Against this backdrop of attenuated classical markers, deletion of 17p13.1 emerges as a notable exception [40]. Rather than fading under therapeutic pressure, *TP53* loss appeared to maintain or even increase its prevalence across disease categories. In chronic lymphocytic leukemia, del(17p) was observed in 15.4% of the diagnostic subset, a figure that exceeds the 7–10% expected in treatment-naïve cohorts and suggests either referral bias toward high-risk patients at an academic center or the early acquisition of this lesion during disease evolution [40]. In multiple myeloma, the observed 11.1% fell within the expected 10–15% range, while in myelodysplastic syndromes it remained below benchmark at 2.2%. The transdiagnostic distribution of del(17p) across multiple disease categories in the association matrix suggests that this lesion may represent a shared adverse-risk feature across hematologic malignancies rather than a disease-specific abnormality [41]. The appearance of this lesion in previously negative patients during longitudinal surveillance highlights its potential relevance during disease evolution and warrants further investigation in prospective studies [42].

The longitudinal dimension of this cohort, incorporating serial assessments in longitudinally monitored patients, permits direct observation of clonal evolution over time. The Sankey flows demonstrate a clear narrative: while the proportion of patients with no detectable aberration remained stable, individual lesions such as del(13q), del(5q), and del(17p) showed progressive attrition, their place taken by an expanding category of rare or complex abnormalities that rose from 16.3% to 36.7% of the cytogenetic landscape. The diversification is compatible with progressive clonal evolution and increasing cytogenetic heterogeneity over time; however, the present retrospective dataset does not allow attribution of these changes to specific therapeutic or biological mechanisms, and the observed associations should be interpreted as descriptive rather than causa [40]. The increasing frequency of complex cytogenetic profiles over time is compatible with progressive biological heterogeneity, although causal mechanisms cannot be established in this retrospective analysis.

Several anticipated associations were notably attenuated or absent in our diagnostic subset. IGH rearrangements, which in classical follicular lymphoma cohorts exceed 80% prevalence, showed only a modest association with non-Hodgkin lymphoma overall (φ = 0.18), a finding that reflects the predominance of diffuse large B-cell, mantle cell, and marginal zone histology in our dataset rather than any biological contradiction [42,43]. Similarly, del(11q)/ATM in CLL was observed at only 3.1%, substantially below the 15–20% expected in comprehensive series, suggesting that ATM probe deployment may have been selective rather than universal in routine practice. These gaps remind us that the cytogenetic landscape observed in any given center is not merely a biological given but a product of testing algorithms, referral patterns, and clinical habit.

The principal strengths of this analysis include its large sample size, extended temporal span, and systematic integration of clinical context with genomic data. Yet the limitations are substantial and must be acknowledged. The retrospective, single-center design restricts generalizability; the heterogeneity of FISH probe panels across diseases and time periods introduces variability in detection sensitivity; and the non-random sampling structure, driven by clinical workflows rather than protocolized schedules, invites selection bias that no statistical adjustment can fully expunge. Treatment regimens, response criteria, and survival outcomes were not systematically captured in this retrospective dataset; accordingly, statistical correlation between therapeutic exposure and cytogenetic evolution could not be performed and represents a priority for prospective validation. Consequently, the observed associations between clinical indication and aberration detection may be confounded by unmeasured treatment-related factors, and the results should be interpreted as hypothesis-generating rather than confirmatory.

Future prospective studies incorporating time-dependent covariate analysis will be required to evaluate the impact of de novo cytogenetic acquisition, including del(17p), on survival endpoints. The five-year window captures only the early impact of modern therapeutics, and longer observation will be required to discern whether deep molecular responses ultimately accelerate or delay the emergence of high-risk cytogenetic alterations.

## 5. Conclusions

This study shows that the observed frequency of FISH-detectable cytogenetic abnormalities in routine hematologic practice is strongly associated with clinical context, testing indication, prior treatment history, and sampling time, in addition to the underlying disease category. The lower positivity rates observed for several disease-defining markers compared with historical diagnostic cohorts and the transdiagnostic persistence of *TP53* loss challenge the indiscriminate application of historical prevalence benchmarks to real-world specimens. By quantifying these dynamics and translating them into an exploratory predictive nomogram, we provide a framework for more nuanced interpretation of FISH results in routine clinical practice. The data suggest that cytogenetic findings in hematologic malignancies should be interpreted within their clinical and temporal context, as observed frequencies may vary according to disease stage, indication for testing, and cohort composition.

## Figures and Tables

**Table 1:** Demographic and Clinical Cohort Characteristics. Summary of the comprehensive dataset of 1357 cytogenetic analyses derived from 1092 individual patients, demonstrating the longitudinal nature of hematologic malignancy surveillance with a mean of 1.24 samples per patient.

Characteristic	n	%	Mean ± SD	Median (Range)
Total samples	1357	100	—	—
Unique patients	1092	—	—	1.24 sample/patient (1–6)
Sex				
Male	616	56.4	—	—
Female	476	43.6	—	—
Age (years)			65.9 ± 13.8	68 (13–116)
<40 years	48	4.4	—	—
40–59 years	252	23.1	—	—
60–74 years	486	44.5	—	—
≥75 years	306	27.9	—	—

**Table 2:** Distribution of Hematologic Malignancies and Demographic Stratification. This table delineates the epidemiological architecture of the five-year cohort, demonstrating the predominance of chronic myeloid and lymphoid neoplasms (MDS/MPN, MM, and CLL comprising 75.7% of all analyses) over acute leukemia.

Disease	Samples (n)	% of Total	Unique Patients	Age (Years) Mean ± SD	% Male
MDS/MPN	369	27.2	327	67.5 ± 14.8	57.2
MM	365	26.9	277	67.6 ± 11.0	58.5
CLL	293	21.6	219	68.4 ± 11.8	67.6
NHL	179	13.2	162	62.0 ± 13.3	60.5
CML	88	6.5	82	59.0 ± 15.2	56.1
AML	31	2.3	26	58.3 ± 20.1	53.8
Other/Unclassified	32	2.4	32	60.8 ± 19.2	58.0
Total	1357	100	1092	65.9 ± 13.8	56.4

**Table 3:** Age Distribution Summary Statistics (Quartiles and Range) Across Disease Entities. This table provides the five-number summary for age distributions, revealing the interquartile ranges and extreme values.

Disease	Min	25th Percentile	Median	75th Percentile	Max
AML	17	32	64	80	80
CML	18	48	59	71	86
NHL	14	52	62	73	116
MM	28	60	69	76	86
CLL	33	60	70	77	91
MDS/MPN	13	64	71	80	95

**Table 4:** Multivariable Logistic Regression Analysis of FISH Aberration Detection.

Variable	Reference Category	Adjusted OR	95% CI	*p*-Value
Clinical indication				
Diagnostic	(ref)	1	—	—
Follow-up	—	0.48	0.36–0.64	<0.001
Relapse	—	1.82	1.28–2.59	0.001
Age (per 10 years)	—	1.24	1.12–1.38	<0.001
Sex (Male vs. Female)	—	1.12	0.89–1.41	0.33
Disease (vs. MDS/MPN)				
CLL	—	2.84	2.12–3.81	<0.001
MM	—	1.35	1.02–1.79	0.036
NHL	—	1.92	1.28–2.88	0.002
CML	—	0.62	0.41–0.94	0.024

**Table 5:** Prevalence of FISH-Detectable Cytogenetic Aberrations Among Evaluated FISH Assays Across Disease Entities.

Disease	Analyzed FISH Assays	Normal n (%)	Abnormal n (%)	95% CI for Abnormality
CLL	276	151 (54.8)	125 (45.3)	39.1–51.4%
NHL	168	112 (66.7)	56 (33.3)	26.2–41.0%
MM	312	218 (69.9)	94 (30.1)	24.9–35.6%
MDS/MPN	488	388 (79.5)	100 (20.5)	16.6–24.6%
CML	82	68 (82.9)	14 (17.1)	9.7–26.8%
AML	31	24 (77.4)	7 (22.6)	9.6–41.1%
Total	1357	961 (70.8)	396 (29.2)	26.8–31.6%

**Table 6:** Comparison of Observed FISH Positivity Rates vs. Literature-Reported Diagnostic Prevalences of Selected Cytogenetic Aberrations. Real-world prevalence estimates derived from the diagnostic cohort (n = 857) are contrasted with established diagnostic benchmarks from the literature. Values represent the percentage of informative FISH assays within each disease category. Notable attenuations include BCR::ABL1 in CML (20.6% vs. >95%), del(13q14) in CLL (24.8% vs. 50–55%) and MM (11.1% vs. 40–50%), and del(11q)/ATM in CLL (3.1% vs. 15–20%). Conversely, del(17p) in CLL (15.4% vs. 7–10%) exceeded expected ranges, while trisomy 12 in CLL (13.0% vs. 10–20%) fell within expectations. Observed rates reflect positivity among informative assays performed in routine practice and should not be interpreted as biological prevalence estimates in treatment-naïve populations.

Aberration	Disease	Observed	Expected	n Tested
BCR-ABL1	CML	20.6%	>95%	68
del(13q14)	CLL	24.8%	50–55%	161
del(13q14)	MM	11.1%	40–50%	190
del(17p)	CLL	15.4%	7–10%	162
del(17p)	MM	11.1%	10–15%	189
del(17p)	MDS/MPN	2.2%	5–10%	232
del(5q)	MDS/MPN	13.2%	10–15%	235
del(7q)/−7	MDS/MPN	3.4%	15–20%	234
Trisomy 12	CLL	13.0%	10–20%	161
del(11q)/ATM	CLL	3.1%	15–20%	161

## Data Availability

The data that support the findings of this study are available on request from the corresponding author.

## Supplementary Materials

The following supporting information can be downloaded at: https://www.mdpi.com/article/10.3390/genes17080955/s1, Supplementary Figure S1: Model calibration and internal validation. Model calibration was assessed using the Hosmer–Lemeshow test (χ^2^ = 8.14, df = 8, p = 0.42), showing good agreement between predicted probabilities and observed outcomes. Supplementary Figure S2: Calibration plot of the prediction model. The calibration plot showed close agreement between the observed event frequencies and the corresponding predicted probabilities across the full range of predicted risk, with the observed values closely tracking the diagonal line representing perfect calibration. No consistent pattern of overestimation or underestimation of risk was evident. Table S1: Patient-level mapping of the study cohort. The table provides a complete patient-level mapping of the study cohort, including the clinical indication, disease category, and identification of patients who contributed multiple samples to the analysis. Supplementary Table S2. Distribution of specimens and longitudinal sampling. The table provides the complete distribution of specimens included in the study, with patient-level identifiers and longitudinal sampling patterns for individuals who underwent multiple assessments over time.

## Abbreviations

The following abbreviations are used in this manuscript:

AML	Acute myeloid leukemia
CLL	chronic lymphocytic leukemia
CML	chronic myeloid leukemia
FISH	Fluorescence in Situ Hybridization
MDS	Myelodysplastic syndromes
MM	Multiple myeloma
NHL	Non-Hodgkin lymphoma
